# Dog Owners' Perspectives on Canine Dental Health—A Questionnaire Study in Sweden

**DOI:** 10.3389/fvets.2020.00298

**Published:** 2020-06-09

**Authors:** Karolina Brunius Enlund, Carl Brunius, Jeanette Hanson, Ragnvi Hagman, Odd Viking Höglund, Pia Gustås, Ann Pettersson

**Affiliations:** ^1^Department of Clinical Sciences, Swedish University of Agricultural Sciences, Uppsala, Sweden; ^2^Anicura Albano Animal Hospital, Stockholm, Sweden; ^3^Department of Biology and Biological Engineering, Food and Nutrition Science, Chalmers University of Technology, Gothenburg, Sweden

**Keywords:** dog, survey, dental health, periodontal disease, breeds

## Abstract

Periodontal disease is one of the most common diseases affecting dogs, with a reported prevalence of at least 80% in dogs over 3 years of age. However, there is a lack of studies regarding dog owners' assessment of their dog's dental health, and whether they perceive clinical signs often associated with periodontal disease, i.e., dental calculus, halitosis or mobile or lost teeth. A validated questionnaire survey was distributed to all Swedish dog owners with email addresses in the national registry (*n* = 209,263). The response rate was 32%. The survey questions concerned opinions and practices regarding canine dental health, including assessment of dental health parameters and dog owners' ability to examine their dog's mouth. A construct (α = 0.76) was used to investigate dog owners' assessed symptoms of their dog's dental health in relation to background factors. Half of the respondents rated their dog's dental health as very good. However, one in four dog owners experienced difficulties when inspecting the dog's teeth. The most common reason for this difficulty was stated to be an uncooperative dog. Almost half of the dog owners reported halitosis to some degree in their dog, and almost four in ten owners reported dental calculus. One in eight dogs had been previously anesthetized for dental cleaning, and one in 12 dogs had experienced problems with gum disease, according to the owners. Owners' assessment varied significantly with the dog's age, weight, breed, breed group, sex, and concurrent disease. Owner-related factors that influenced the assessment of the dog's dental health were age, gender, education, county (urban/rural), and whether they were breeders or not. Dog owners with smaller dogs, older dogs and certain breeds predisposed to periodontal disease assessed their dog's dental health as worse than their counterparts, which is in agreement with previously reported higher prevalence of dental disease in these groups. This indicates that dog owners are able to perform relative assessment of their dog's dental health status. Our results also highlight the need for routine professional assessment of periodontal health, as well as education of dog owners and training of dogs to accept dental care procedures.

## Introduction

Although one of the most common diseases affecting dogs, with a reported prevalence of 80–89% in dogs over 3 years of age (1–4), periodontal disease is often overlooked and may therefore be inadequately treated and prevented (5). Periodontal disease is an inflammatory disease affecting the tooth supporting tissues which may lead to progressive tissue and tooth loss (6). Studies have shown an increased prevalence of periodontal disease in smaller dogs, and the severity of the disease in general increases with age (1, 7, 8). Today, the potential systemic effects of periodontal disease is of growing concern and a multitude of studies have been presented within human medicine, identifying associations between periodontal disease and other diseases, such as diabetes mellitus, cardiovascular disease, and immunological disease (9, 10). In veterinary medicine, however, few studies have as yet been published on the topic and some have shown conflicting results, indicating the need for further standardized prospective studies within this field (5, 11–13).

Owners' different ability to examine their dog's mouth and their knowledge concerning normal and pathologic dental conditions may considerably influence their assessment of their pet's dental health. Dog owners may or may not recognize clinical signs of periodontal disease, e.g., halitosis, gingival inflammation and recession, and tooth mobility or tooth loss. The presence of dental calculus, visible to dog owners, is not indicative of periodontal disease *per se*, although it may indicate poor dental hygiene. Experts agree that a thorough dental exam, including periodontal probing of all tooth surfaces, and intraoral radiographic examination, is necessary to assess clinical attachment loss, i.e., degree of periodontal disease. Consequently, even veterinarians may be unable to assess dental health properly during the clinical examination of a non-anesthetized animal (5). This highlights the need for professional dental assessment under anesthesia on a regular basis for all dogs.

Daily tooth brushing is considered the gold standard for prevention of periodontal disease development and progression (14–18). Compliance to the recommendation of daily tooth brushing in veterinary patients is low (19). This may, in part, be explained by a lack of knowledge regarding dental disease (19), and an important factor to increase compliance may thus be to increase awareness of clinical signs of disease among dog owners. Additionally, dental home care is not possible without the dog owner being able to handle the dog's mouth, which requires a certain amount of skill and training of both dog and owner. However, studies regarding whether, and how, dog owners assess their dog's dental health are lacking.

Within the framework of a nationwide survey on canine dental home care (19, 20), we have investigated canine dental health from a dog owner perspective. If properly constructed and validated, questionnaire surveys provide a useful method for evaluating attitudes, opinions and practices on specific topics (20–22).

The aim of the present study was to investigate dog owners' general opinions, as well as their assessment, of their dog's dental health. Additionally, associations between perceived dental problems and specific non-dental chronic diseases were explored.

To our knowledge, this is the first survey presented with this objective.

## Materials and Methods

### Study Design

In order to investigate dog owners' opinions and assessment of their dog's dental health, and perceived dental problems and specific non-dental chronic diseases, a questionnaire survey to dog owners was analyzed. This was part of a large study including questionnaire surveys to dog owners, veterinarians and veterinary nurses, which were constructed and validated according to survey methodology guidelines, as described elsewhere (19, 20). The target group consisted of all registered dog owners in Sweden, 607,610 individuals. Sample frames were dog owners with email addresses registered with the Swedish Board of Agriculture (13 March 2017) and email addresses registered with the Swedish Kennel Club (9 February 2017), giving in total 209,263 email addresses of dog owners.

The questionnaire survey was adapted for use on personal computers, tablets and smartphones, using the web platform Netigate (Netigate AB, Stockholm, Sweden). The questionnaires were distributed and reminders were sent to non-responders after 8 and 17 days. Data collection started on 31 March and was completed on 30 April 2017. Anonymous responses were collected and the questionnaire could only be answered once per link. If the household owned more than one dog (23% owned more than one dog, personal communication, Magnus Kindström, Swedish Board of Agriculture, 28 August, 2017), the respondent was asked to choose one of them and answer for the same dog throughout the survey (20). The study was approved by the Regional Ethical Review Board in Uppsala (Dnr 2017/035).

The total length of the questionnaire for dog owners ranged from 54 to 68 questions for the individual respondents depending on their answers. The questions were mainly closed, i.e., with fixed response options, and both nominal and ordinal data were collected (20). Twelve questions [Questions 5, 6, 7, 18, 19, 20, 23, 24, 25, 26, 28, 29 (20)] concerned owners perception of the dog's dental and general health. In particular, questions 5, 6, and 7 relate to dog owners' general opinions on the dental health of their dog, and questions 19 and 20 to their assessment of the dental health of their dog, questions 18, 23, 24, 25, and 26 to their dogs dental health, and questions 28 and 29 to general health and specific non-dental chronic diseases.

### Statistical Analysis

Responses to survey questions are reported as percentages rounded to the nearest first decimal, and may consequently not sum up to exactly 100%.

Pretreatment of data, including identification and validation of constructs, is described in detail elsewhere (20). In brief, exploratory factor analysis (EFA) was performed on random half-splits of numeric and ordinal non-sociodemographic data to identify factors, which were confirmed in the other half-split using confirmatory factor analysis (CFA). Final construct scores were extracted from CFA on all data using variables selected from the EFA/CFA validation procedure. The construct used in this study “Dog owners' assessed symptoms of their dog's dental health” reflected core concepts regarding canine dental health and these are illustrated in Table 1.

**Table 1:** Questions included in the construct: “Dog owners' assessed symptoms of their dog's dental health” (α = 0.76) (20).

How would you appraise your dog's dental health? (Q5)
Has your dog been anesthetized at a veterinary clinic to clean the teeth/remove dental calculus? (Q23)
Has your dog had problems with gum disease or loose teeth? (Q24)
Does your dog have bad breath? (Q25)
Does your dog have dental calculus at the moment? (Q26)
How would you appraise your dog's general health? (Q28)

*Numbers in parentheses correspond to the number of the question in the full survey (20)*.

All statistical analysis was performed in the R open source statistical software v 3.5.1 (23). Overall significance of fixed factors in linear mixed modeling was assessed by type III tests and using Tukey adjustment for pairwise comparisons. Results are reported as least squares means with 95% CI. Results from logistic regressions are reported as odds ratios with 95% CI.

The breeds were grouped into 10 breed groups as used by the FCI (Federation Cynologique Internationale) (24) as well as the Swedish Kennel Club, for further analysis (Table 2).

**Table 2:** Breed groups, adapted from FCI (Federation Cynologique Internationale) (24).

Group 1	Sheepdogs and Cattledogs (except Swiss Cattledogs)
Group 2	Pinscher and Schnauzer—Molossoid and Swiss Mountain and Cattledogs
Group 3	Terriers
Group 4	Dachshunds
Group 5	Spitz and primitive types
Group 6	Scent hounds and related breeds
Group 7	Pointing Dogs
Group 8	Retrievers—Flushing Dogs—Water Dogs
Group 9	Companion and Toy Dogs
Group 10	Sighthounds


The Dental Health construct was analyzed by linear mixed modeling using the R “glm” function. Dog weight group, sex and breed group, reported diseases (Figure S1d) and owner gender, level of education, county (urban vs. rural), employment, medical profession (assistant nurse, nurse, physician, dental nurse, dental hygienist, dentist, animal caretaker, veterinary nurse/technician, veterinarian) and breeder status were included as fixed factors. In addition, dog and owner year-of-birth were added as covariates. To investigate the association between Dental Health and breeds, a linear model was used which included dog breed and sex as well as owner gender, level of education, county (urban vs. rural), employment, medical profession and breeder status as fixed factors, and dog and owner year-of-birth as covariates.

The questions “How would you appraise your dog's general health?” (Q28), “How important is it for you that your dog has good dental health?” (Q6) and “How easy or difficult is it for you to inspect (look at) all of your dog's teeth?” (Q19) were analyzed by ordinal logistic regression using the R'polr' function from the “MASS” package and with the same fixed factors and covariates.

## Results

The total number of respondents was 66,434, corresponding to a response rate of 32%. After removing individuals with >20% missing data among selected background questions, there were a total of 59,978 completed individual responses (20).

Background characteristics of dog owner respondents and their dogs are described in detail elsewhere (19). In brief, the dogs were 4.9 ± 3.5 years of age (mean ± SD). All breed groups were represented. Breed group 8 (Retrievers, Flushing Dogs, Water Dogs) was the largest (18%), followed by dogs of mixed breed (15%), and Group 9 (Companion and Toy Dogs) (15%). German Shepherd Dog, Labrador Retriever and Golden Retriever were the most common pure breeds, comprising more than 10% of all dogs (Figure S1e). One-third (33%) of dogs weighed under 10 kg and the majority (78%) of all dogs were sexually intact (19).

Dog owners were 49.9 ± 13.4 years of age (mean±SD), 74.8% were women, 24.6% were men and 0.7% preferred not to answer the question or defined themselves as other. Forty six percent of all dog owners lived in urban counties (Stockholm, Skåne, Västra Götaland). Seventy percent were employed or self-employed. Forty nine percent had studied at a university and 23% reported that they worked within a healthcare profession. Moreover, one in twelve (8%) was a dog breeder (19).

**Figure 1:**
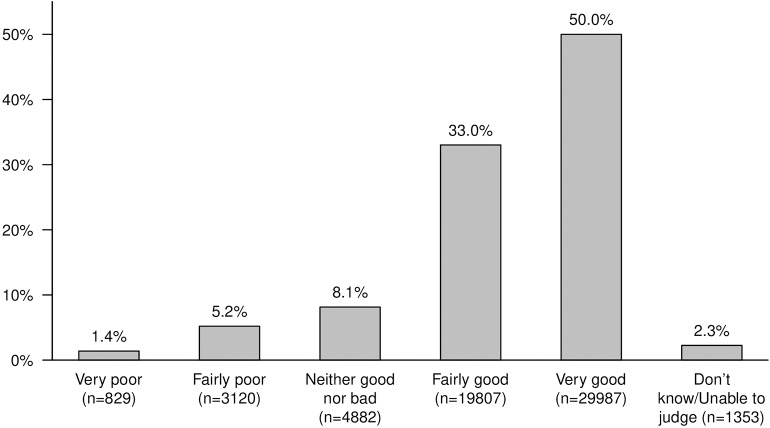
How would you appraise your dog's dental health?

**Figure 2:**
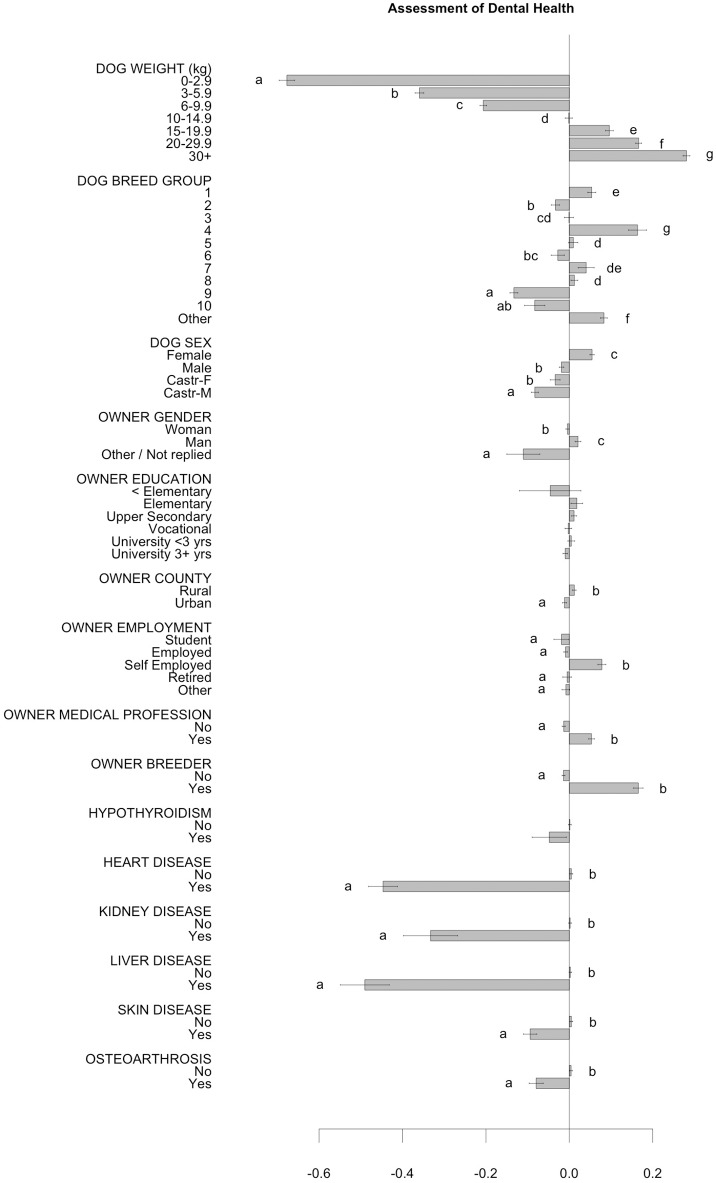
Dog owners' assessed symptoms of their dog's dental health. Associations between background characteristics of dog/dog owner, and dog owners' assessed symptoms of their dog's dental health. Higher construct score represents a relatively better perceived dental health. Scores should only be compared within figure. Note that negative scores do not automatically reflect a negative assessment of dental health.

**Figure 3:**
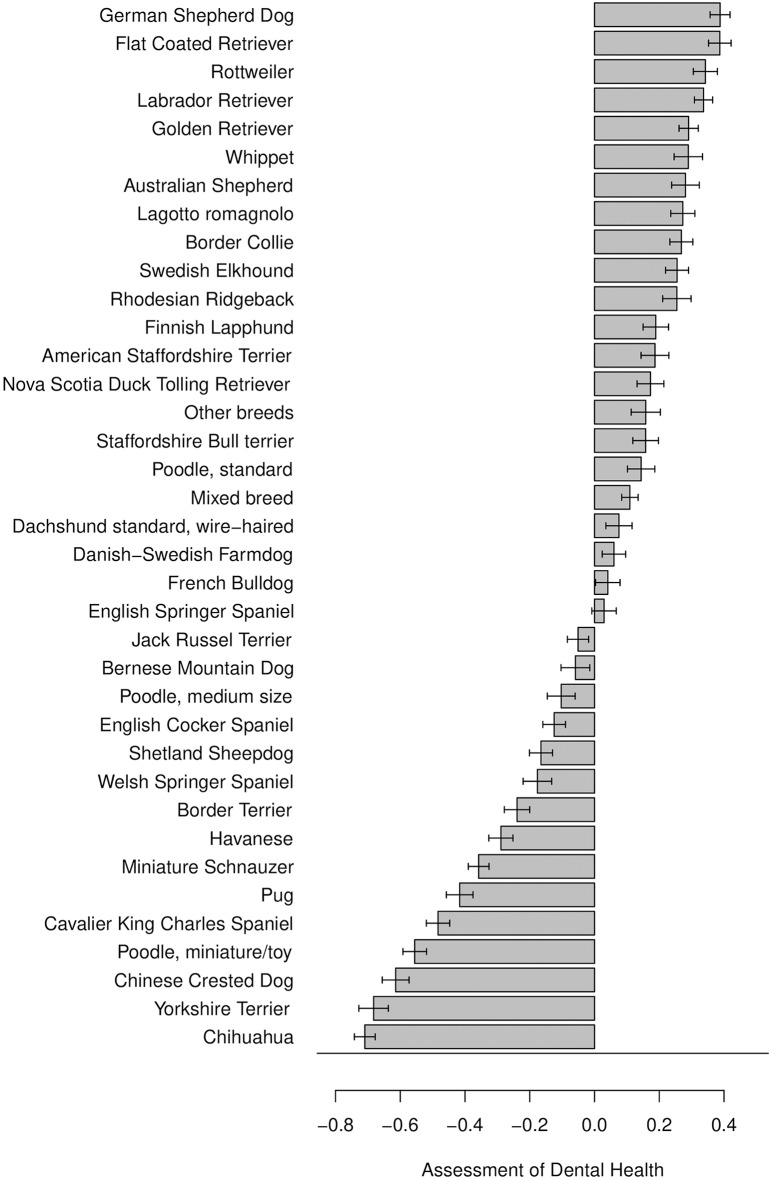
Association of breeds with owners' assessed symptoms of their dog's dental health. Reported for breeds with ≥400 respondents. Higher construct score represents a relatively better perceived dental health. Scores should only be compared within figure. Note that negative scores do not automatically reflect a negative assessment of dental health.

Survey results are summarized in Tables 3, 4 and Figures 1–**3**. Seventy eight percent of owners perceived their dog's general health as very good (Table 3), and half (50%) stated their dog's dental health as very good (Figure 1). Of the owners of dogs over 3 years of age, 38% rated dental health as very good. The owners of dogs in breed group 9 (Companion and Toy Dogs) rated their dog's general health lowest, and owners of dogs in breed group 7 (Pointing Dogs) highest. Survey participants living in urban counties and owners of younger dogs rated their dog's general health as better than those living in rural counties and owning older dogs (Figure S1a).

**Table 3:** Results from the survey.

Has your dog been anesthetized at a veterinary clinic to clean the teeth/remove dental calculus? (Q23)	No	Yes, once			Yes, several times	Don't know
	51,789 (86.3%)	5,217 (8.7%)			2,620 (4.4%)	352 (0.6%)
						
Has your dog had problems with gum disease or loose teeth? (Q24) (Does not apply to puppy teeth)	No	Yes, has had teeth extracted or lost teeth			Yes, but has not had any teeth extracted	Don't know
	55,033 (91.8%)	3,900 (6.5%)			729 (1.2%)	316 (0.5%)
						
Does your dog have bad breath? (Q25)	No, never	Yes, sometimes	Yes, often	Yes, always	Don't know
	31,033 (51.7%)	24,427 (40.7%)	2,961 (4.9%)	1,275 (2.1%)	282 (0.5%)
Does your dog have dental calculus at the moment? (Q26)	No	Yes, a little	Yes, a moderate amount	Yes, a lot	Don't know
	31,159 (52.0%)	18,753 (31.3%)	2,925 (4.9%)	673 (1.1%)	6,468 (10.8 %)
How would you appraise your dog's general health? (Q28)	Very poor	Fairly poor	Neither good nor bad	Fairly good	Very good	Don't know/Unable to judge
	196 (0.3%)	565 (0.9%)	1,229 (2.1%)	11,102 (18.5%)	46,715 (78.0%)	102 (0.2%)

*Questions from the construct: “Dog owners' assessed symptoms of their dog's dental health.” Percentages are rounded to the nearest first decimal. Number in parenthesis after the question corresponds to the number of the question in the full survey (20)*.

**Table 4:** Results from the survey.

How important is it for you that your dog has good dental health? (Q6)		Not at all important	Of minor importance	Fairly important	Very important	Don't know
		18 (0.0%)	267 (0.4%)	11,354 (18.9%)	48,089 (80.2%)	250 (0.4%)
What do you consider to be important for good dental health in dogs? (Q7)	Good general health	43 (0.1%)	197 (0.3%)	8,319 (14.5%)	48327 (84.4%)	342 (0.6%)
	The dog's breed/heredity	1,230 (2.2%)	5,337 (9.3%)	23,400 (41.0%)	18,033 (31.6%)	9,093 (15.9%)
When you clean your dog's teeth at home, does the gum ever bleed? (Q18) *(Only visible to respondents who brush/clean their dog's teeth)*		No, never	Yes, sometimes	Yes, often	Yes, always	Don't know
		19,171 (60.6%)	10,107 (32.0%)	684 (2.2%)	165 (0.5%)	1,489 (4.7%)
					
How easy or difficult is it for you to inspect (look at) all of your dog's teeth? (Q19)		Very easy	Fairly easy	Fairly difficult	Very difficult	Don't know
		22,146 (36.9%)	22,897 (38.2%)	11,399 (19.0%)	3,270 (5.5%)	266 (0.4%)
What difficulties do you experience when inspecting your dog's teeth? (Q20) Several options can be specified *(Only visible to respondents who answered “Fairly” or “Very difficult” on Q19)*		The dog is in pain The dog gets angry The dog doesn't want to Own impaired physical ability I do not know how to do it Technically/practically difficult to perform Don't know Other reason			82 (0.6%) 1,103 (7.5%) 11,609 (79.1%) 303 (2.1%) 1,318 (9.0%) 4,642 (31.6%) 139 (0.9%) 400 (2.7%)	

*Questions not included in the construct “Dog owners' assessed symptoms of their dog's dental health.” Percentages are rounded to the nearest first decimal. Number in parenthesis after the question corresponds to the number of the question in the full survey (20)*.

Among breeds with ≥ 100 respondents per breed, owners of Briard (78%), Dobermann (77%), and Giant Schnauzer (76%) were most likely to answer that their dog had very good dental health. The owners of a Prazský krysarík (19%), Chinese Crested Dog (25%), Pomeranian (25%), Italian Greyhound (25%), and Chihuahua (27%) dogs were least likely to report very good dental health. The owners of Pug (34%), Chihuahua (21%), Yorkshire Terrier (17%), Pomeranian (16%) and Papillon (16%) dogs were most likely to report it very difficult to inspect the dog's teeth.

Four out of five (80.2%) owners considered the dog's dental health to be very important (Table 4). Owners of dogs over 30 kg considered the dental health of their dog more important than owners of smaller dogs did. Men were less likely than women (OR = 0.55; 95%CI: 0.53–0.58) to consider dental health in their dog important. Dog breeders and owners from urban counties considered dental health more important than owners from rural counties did, and dog owners with a higher education level considered dental health of their dog less important than owners with a lower education level (Figure S1b). 84.4% of dog owners regarded good general health as very important for good dental health, and the dog's breed (heredity) was considered to be very important by 31.6% of dog owners (Table 4).

One in four (24.5%) owners sometimes or always experienced difficulties when inspecting the dog's teeth (Table 4), and the smaller the dog, the more difficult (Figure S1c). Breeders and owners from urban counties found it easier to inspect their dog's teeth than owners from rural counties. The owners of dogs in breed group 9 (Companion and Toy Dogs) found it most difficult, and owners of dogs in breed group 4 (Dachshunds) and 7 (Pointing Dogs) found it least difficult to inspect the dog's teeth (Figure S1). The most common reasons for these difficulties were stated as an uncooperative dog (79.1%) and practical/technical difficulties (31.6%) (Table 4).

47.7% of the dog owners reported halitosis to some degree, and 37.3% of owners reported the presence of dental calculus. Of the owners who cleaned their dog's teeth (19), 34.7% stated occasional oral bleeding (Table 4). 13.1% of dogs had been previously anesthetized for dental cleaning, and 7.7% of dogs had experienced problems with gum disease/mobile teeth, according to the owner (Table 3).

Associations between background characteristics of dogs and dog owners, and dog owners' assessed symptoms of their dog's dental health are shown in Figures 2–3 and in Figure S1f. Reported construct scores of “Dog owners' assessed symptoms of their dog's dental health” were lower, indicating worse perceived dental health, the smaller the dog was (Figure 2). The owners (*n* > 400 respondents/breed) of German Shepherd Dog, Flat coated Retriever and Rottweiler had the highest scores in the construct, reflecting a perceived better dental health than owners of Chihuahua, Yorkshire Terrier and Chinese Crested Dog, who had the lowest scores (Figure 3). Women perceived dental health in their dog as worse than men did, and breeders perceived dental health as better than their counterparts (Figure 2). Younger dog owners and owners of older dogs perceived their dog's dental health as worse than their counterparts (Figure S1f).

The most common concurrent diseases, among the alternatives provided in the questionnaire, were skin disease (3.9%), and joint disease (3.7%) (Figure S1d). Concurrent diseases reported by ≥100 dog owners were investigated further and were associated with a more negative dental health assessment, in particular for cardiac disease, renal disease and hepatic disease (Figure 2).

Odds ratios for the association of dogs'/dog owners' background characteristics with dog owners' rated general health, stated importance of dental health, and stated difficulties in inspecting the dog's teeth, are shown in Supporting Information (Figures S1a–c).

## Discussion

This study reports the results from a questionnaire to dog owners regarding their dog's dental health. The vast majority of participating dog owners responded that the dental health of their dog was important. In addition, the huge interest and dedication of the respondents, with more than 66,000 individual respondents and almost 9,000 free text comments, clearly showed dog owners' engagement in the dental health of their dog, which was an important finding in itself.

### General and Dental Health

Most dog owners, almost eight out of 10, regarded the general health of their dog to be very good, while only half of the dog owners perceived their dog's dental health to be at the same high level. One third of dog owners rated their dog's dental health as only fairly good, indicating that they had, in fact, noted a deviation from an optimal situation. However, this overall positive assessment of dental health is in contrast to the veterinarians' and veterinary nurses' estimations of dental problems as very or fairly common (19), and with the known high prevalence of canine dental disease (1–3). Several explanations for this discrepancy are possible. First, there is likely a lack of knowledge among dog owners concerning periodontal disease and its clinical signs. Also, there may be difficulties to thoroughly examine the dog's teeth, as reported in the present survey. Since dogs do not often show apparent signs of dental discomfort, owners are likely to underestimate dental problems as well as their impact on general well-being. Furthermore, there is an inherent lack of precision in the terminology. For example, where an owner may observe a minor dental problem and assess dental health as “Fairly good,” a veterinary health professional may upon clinical examination find signs of periodontal disease. Finally, anesthesia is required to fully examine a dog's dental status, including clinical attachment loss, which means that oral examination in the awake animal may be insufficient in the majority of cases for a full diagnosis.

The fact that more than one in four dog owners stated that they did not know if breed was an important factor related to dental health, or stated that it was not important, highlights a potential knowledge gap among dog owners, since the predisposition of some breeds to periodontal disease is well-known (2, 25–28).

### Dog Owners Assessment of Their Dog's Dental Health

Discouragingly, as many as one in four dog owners experienced difficulties when inspecting the dog's teeth. If dog owners cannot manage to examine the mouth properly, it is also likely that tooth brushing is difficult or even impossible. The most common reported reasons for these difficulties were uncooperative dogs and practical/technical difficulties, highlighting the need for early training of both dogs and owners in dental home care routines.

In spite of the known high prevalence of canine dental disease, only 13% of dogs in this study had been previously anesthetized for professional dental cleanings and only 6% had lost or extracted teeth, indicating a low number of dental procedures having been performed in this study population. This study is, to the authors' knowledge, the first published investigation on the proportion of privately owned dogs that have undergone dental cleaning under anesthesia. In comparison, despite anesthesia-free dentistry being strongly discouraged by the veterinary dental community (5), we have previously reported that more than 20% of the dog owners in our study population reported the use of a dental scaler to remove calculus on a non-anesthetized dog (19). Although the study population was relatively young, with about 45% of dogs being 3 years old or less (19), these findings in conjunction with the high reporting of halitosis [which is most commonly caused by dental disease (29)], suggest that what is presented at the clinic is only the tip of the iceberg, leaving many dogs with untreated or incorrectly treated dental problems.

We cannot know the actual amount of dental calculus in the dogs participating in the study. However, almost four out of ten dog owners made the assessment that their dog had dental calculus to some degree (Table 3). It is more likely that the amount of calculus is underestimated than overestimated. In addition, it is remarkable that more than one out of ten owners did not know if their dog had dental calculus. These results are likely a consequence of dog owners' lack of ability to correctly identify dental calculus and also to properly inspect the dog's teeth.

Gingival bleeding is an indication of gingivitis in both humans and in dogs. More than one third of dog owners reported oral bleeding when brushing (Table 4), and a comparison with previously published results (19) showed that brushing less frequently is associated with increased risk of bleeding (χ^2^-test *p* <2.2 × 10–16). Also considering that almost one third of Swedish dog owners stated that they brushed more seldom than once a week (19), the low frequency of brushing has likely led to persistent gingivitis. No other questions regarding gingivitis were included in the study, due to the risk of incorrect owner assessment of gingival inflammation and/or recession.

The fact that owners' perception of their dog's dental health varied with breed, age, and weight is in accordance with the previously reported higher prevalence of periodontal disease in smaller and older dogs as well as in particular breeds (1, 2, 7, 25–28). A previous study in humans has shown low reliability of self-evaluation of periodontal variables (30). However, a significant positive correlation between dog owners' and veterinarians' assessment of dental health, in non-anesthetized dogs, was recently shown (31). Together, these studies indicate that dog owners' reports on perceived dental problems are likely true, whereas non-reporting cannot be seen as an absence of disease. Moreover, in the present study the dog's dental health was generally considered good, contrary to the known high prevalence of dental disease reported in other studies (1–3, 7, 8). The fact that one in four dog owners experienced difficulties inspecting the dog's teeth may also contribute to the underreporting of problems. Our conclusion is that owners seem capable of identifying dental problems, although the true extent of dental disease is likely underestimated.

### Other Diseases

The survey also investigated owner-reported prevalence of some common non-dental chronic diseases, known to have immunological /inflammatory properties (Cushing's disease, Addison's disease, hypothyroidism, skin disease/allergy, osteoarthrosis) or to have more direct associations with periodontal disease in humans and/or dogs (diabetes, cardiac disease, hepatic disease, renal disease) (11, 12, 32, 33). Especially, owner-reported cardiac, hepatic and renal disease were associated with worse assessed dental health. Dental health may be affected by the concurrent disease or medication, or problems may be acknowledged to a higher degree, for example because of increased owner awareness. Associations between worse periodontal health and numerous diseases have also been found in humans. However, causality is not evident and mechanisms describing the relationship between general health and dental health remain to be elucidated (9, 10, 34).

### Strengths and Limitations

In order to ensure high data quality, it is essential to construct surveys according to evidence based methods and validation procedures. The construction and validation of the survey used in this study have previously been presented in detail. The validation further showed that the representatively of the respondents was satisfactory overall (20). The large study sample in this study constitutes a major strength, ensuring that the obtained data are likely to give a correct reflection of the enquired opinions and attitudes.

Despite meticulous efforts to avoid bias, questionnaire surveys are inevitably susceptible to recruitment bias, social desirability bias, and acquiescence bias. A potential risk is that respondents may be more interested in the subject than the average population. Further, misinterpretation of preformulated answers is always a potential risk. As a part of the questionnaire validation process, efforts were made to limit the use of vague response options such as “sometimes”/ “often” or “very good”/“very poor.” In this study, however, the objective was not to measure actual frequencies, but instead to examine opinions, necessitating the use of the more vague response alternative (20).

Clinical examinations were not performed and dental health assessments made by the dog owners could, consequently, not be validated. However, the construct had high internal consistency and high reliability, as assessed by scientific and clinical experts (20). In addition, the construct confirmed known associations between dental health and dog breed, weight, and age, indicating high construct validity (20).

Another limitation of the present study regarded the diagnosis of non-dental diseases. Some of these (e.g., Cushing's disease, Addison's disease, hypothyroidism) were likely based on the actual diagnosis made by a veterinarian, but other medical problems (e.g., skin disease/allergy, ostheoarthrosis) may have only been based upon owner's perception.

## Conclusion

Dog owners with smaller dogs, older dogs, and certain breeds known to be predisposed to periodontal disease, assessed their dog's dental health as worse than their counterparts, which is in agreement with previously reported higher prevalence of dental disease in these groups. This indicates that dog owners are able to perform relative assessment of their dog's dental health status.

The known high prevalence of dental disease, together with the low reported frequency of professional dental cleaning under anesthesia, highlights the need for routine professional assessment of periodontal health and education of dog owners on the importance of dental care. Dog owners' difficulties in inspecting their dog's teeth underline the need for education of dog owners and training of dogs to accept dental home care procedures.

## Data Availability Statement

The data is available from the authors upon reasonable request. The data are not publicly available due to them containing information that could compromise research participant privacy.

## Ethics Statement

The studies involving human participants were reviewed and approved by the Regional Ethical Review Board in Uppsala (Dnr 2017/035). Written informed consent for participation was not required for this study in accordance with the national legislation and the institutional requirements.

## Author Contributions

KE writing: original draft and study design. CB statistical analysis, writing: review and editing. JH, RH, OH, and PG: writing: review and editing. AP conceptualization, study design, writing: review and editing. All authors read and approved the final manuscript.

## Conflict of Interest

The authors declare that the research was conducted in the absence of any commercial or financial relationships that could be construed as a potential conflict of interest.

## References

[1] Hamp SE Olsson SE Farsomadsen K Viklands P Fornell J A macroscopic and radiologic investigation of dental diseases of the dog. Vet Radiol. (1984) 25:86–92. 10.1111/j.1740-8261.1984.tb01916.x

[2] Kortegaard HE Eriksen T Baelum V. Periodontal disease in research beagle dogs–an epidemiological study. J Small Anim Pract. (2008) 49:610–16. 10.1111/j.1748-5827.2008.00609.x 18793256

[3] Fernandes NA Borges APB Reis ECC Sepúlveda RV Pontes KCDS Prevalence of periodontal disease in dogs and owners' level of awareness - a prospective clinical trial. Revista Ceres. (2012) 59:446–51. 10.1590/S0034-737X2012000400003

[4] Stella JL Bauer AE Croney CC. A cross-sectional study to estimate prevalence of periodontal disease in a population of dogs (*Canis familiaris*) in commercial breeding facilities in indiana and illinois. PLoS ONE. (2018) 13:e0191395. 10.1371/journal.pone.0191395 29346448 PMC5773197

[5] WSAVA World Small Animal Veterinary Association Global Dental Guidelines. (2018). Available online at: https://wsava.org/wp-content/uploads/2020/01/Dental-Guidleines-for-endorsement_0.pdf (accessed May 27, 2020).

[6] Niemiec BA Etiology and pathogenesis of periodontal disease. In: Niemiec BA editor. Veterinary Periodontology, Ames, IA: Wiley-Blackwell (2012). p. 18–34. 10.1002/9781118705018.ch2

[7] Harvey CE Shofer FS Laster L. Association of age and body weight with periodontal disease in North American dogs. J Vet Dent. (1994) 11:94–105. 10.1177/089875649401100301 9693607

[8] Kyllar M Witter K Prevalence of dental disorders in pet dogs. Vet Med. (2005) 50:496–505. 10.17221/5654-VETMED

[9] Kim J Amar S. Periodontal disease and systemic conditions: a bidirectional relationship. Odontology. (2006) 94:10–21. 10.1007/s10266-006-0060-6 16998613 PMC2443711

[10] Hajishengallis G. Periodontitis: from microbial immune subversion to systemic inflammation. Nat Rev Immunol. (2014) 15:30–44. 10.1038/nri3785 25534621 PMC4276050

[11] Rawlinson JE Goldstein RE Reiter AM Attwater DZ Harvey CE. Association of periodontal disease with systemic health indices in dogs and the systemic response to treatment of periodontal disease. J Am Vet Med Assoc. (2011) 238:601–9. 10.2460/javma.238.5.601 21355802

[12] Pavlica Z Petelin M Juntes P ErŽen D Crossley DA Skalerič U. Periodontal disease burden and pathological changes in organs of dogs. J Vet Dent. (2008) 25:97–105. 10.1177/089875640802500210 18751659

[13] Peddle GD Drobatz KJ Harvey CE Adams A Sleeper MM. Association of periodontal disease, oral procedures, and other clinical findings with bacterial endocarditis in dogs. J Am Vet Med Assoc. (2009) 234:100–7. 10.2460/javma.234.1.100 19119972

[14] Harvey C Serfilippi L Barnvos D. Effect of frequency of brushing teeth on plaque and calculus accumulation, and gingivitis in dogs. J Vet Dent. (2015) 32:16–21. 10.1177/089875641503200102 26197686

[15] Tromp JAH Jansen J Pilot T Gingival health and frequency of tooth brushing in the beagle dog model. J Clin Periodontol. (1986) 13:164–8. 10.1111/j.1600-051X.1986.tb01451.x 3455949

[16] Tromp JAH van Rijn LJ Jansen J Experimental gingivitis and frequency of tooth brushing in the beagle dog model. J Clin Periodontol. (1986) 13:190–94. 10.1111/j.1600-051X.1986.tb01458.x 3457807

[17] Gorrel C. Home care: products and techniques. Clin Tech Small Anim Pract. (2000) 15:226–31. 10.1053/svms.2000.21625 11269998

[18] Gorrel C Rawlings JM. The role of tooth-brushing and diet in the maintenance of periodontal health in dogs. J Vet Dent. (1996) 13:139–43. 10.1177/089875649601300405 9520789

[19] Enlund KB Brunius C Hanson J Hagman R Höglund OV Gustås P. Dental home care in dogs - a questionnaire study among Swedish dog owners, veterinarians and veterinary nurses. BMC Vet Res. (2020) 16:90. 10.1186/s12917-020-02281-y 32188446 PMC7081671

[20] Brunius Enlund K Brunius C Hanson J Hagman R Hoglund OV Gustas P. Development and validation of two questionnaires: dental home care and dental health in Swedish dogs. PLoS ONE. (2019) 14:e0204581. 10.1371/journal.pone.0204581 30682017 PMC6347148

[21] Marsden PV Wright JD Handbook of Survey Research. Bingley: Emerald (2010). p. 1–903.

[22] Tourangeau R Rips LJ Rasinski K The Psychology of Survey Response. Cambridge: Cambridge University Press (2000). p. 1–416. 10.1017/CBO9780511819322

[23] R_Core_Team R: A Language and Environment for Statistical Computing. R Foundation for Statistical Computing, Vienna, Austria (2016). Available online at: https://www.R-project.org/ (accessed November 19, 2018).

[24] Federation Cynologique Internationale FCI Breeds Nomenclature. (2019). Available online at: http://www.fci.be/en/Nomenclature/ (accessed February 26, 2019).

[25] Hoffmann T Gaengler P. Epidemiology of periodontal disease in poodles. J Small Anim Pract. (1996) 37:309–16. 10.1111/j.1748-5827.1996.tb02396.x 8840250

[26] Hamp SE Hamp M Olsson SE Lindberg R Schauman P. Radiography of spontaneous periodontitis in dogs. J Periodontal Res. (1997) 32:589–97. 10.1111/j.1600-0765.1997.tb00936.x 9401931

[27] Wallis C Patel KV Marshall M Staunton R Milella L Harris S. A longitudinal assessment of periodontal health status in 53 labrador retrievers. J Small Anim Pract. (2018) 59:560–9. 10.1111/jsap.12870 30006940

[28] Lund E. Using data to understand periodontal disease risk. Banfield J. (2008) 15–20. Available online at: https://www.banfield.com/getmedia/7dbaf3a6-4d63-4693-80cb-b234cd01472d/4_1-Using-data-to-understand-periodontal-disease-risk (accessed May 27, 2020).

[29] Reiter AM Gracis M Dental and oral examination and recording. In: Reiter AM Gracis M editors. BSAVA Manual of Canine and Feline Dentistry and Oral Surgery. Gloucester: British Small Animal Veterinary Association (2018). p. 37 10.22233/20412495.1018.24

[30] Buhlin K Gustafsson A Andersson K Hakansson J Klinge B. Validity and limitations of self-reported periodontal health. Commun Dent Oral Epidemiol. (2002) 30:431–7. 10.1034/j.1600-0528.2002.00014.x 12453114

[31] Aula AM Prevalence of indicators of dental diseases in dogs and cats: risk factors for oral pathology and correlation of owner perception with clinical examination findings. (master's thesis). Eesti Maaülikool. (2018).

[32] DeBowes LJ Mosier D Logan E Harvey C Lowry S Richardson D. Association of periodontal disease and histologic lesions in multiple organs from 45 dogs. J Vet Dent. (1996) 13:57–60. 10.1177/089875649601300201 9520780

[33] Whyte A Bonastre C Monteagudo LV Les F Obon J Whyte J. Canine stage 1 periodontal disease: a latent pathology. Vet J. (2014) 201:118–120. 10.1016/j.tvjl.2014.05.005 24878263

[34] Seymour G Ford P Cullinan M Leishman S Yamazaki K. Relationship between periodontal infections and systemic disease. Clin Microbiol Infect. (2007) 13:3–10. 10.1111/j.1469-0691.2007.01798.x 17716290

