# Supplementary material for: Dog Owners' Perspectives on Canine Dental Health—A Questionnaire Study in Sweden
Source: Front Vet Sci. 2020 Jun 9;7:298. doi: 10.3389/fvets.2020.00298 (PMC7297050; doi:10.3389/fvets.2020.00298)

Dog owners’ perspectives on canine dental health – A questionnaire study in Sweden

Supplementary Figures and Tables

S1a. How would you appraise your dog's general health?

S1b. How important is it for you that your dog has good dental health?

S1c. How easy or difficult is it for you to inspect (look at) all of your dog's teeth?

S1d. Concurrent diseases reported by dog owners.

S1e. The 10 most frequently reported breeds in the target and sample populations.

S1f. Associations of dogs’ and dog owners’ year of birth with owners’ assessed symptoms of their dog’s dental health

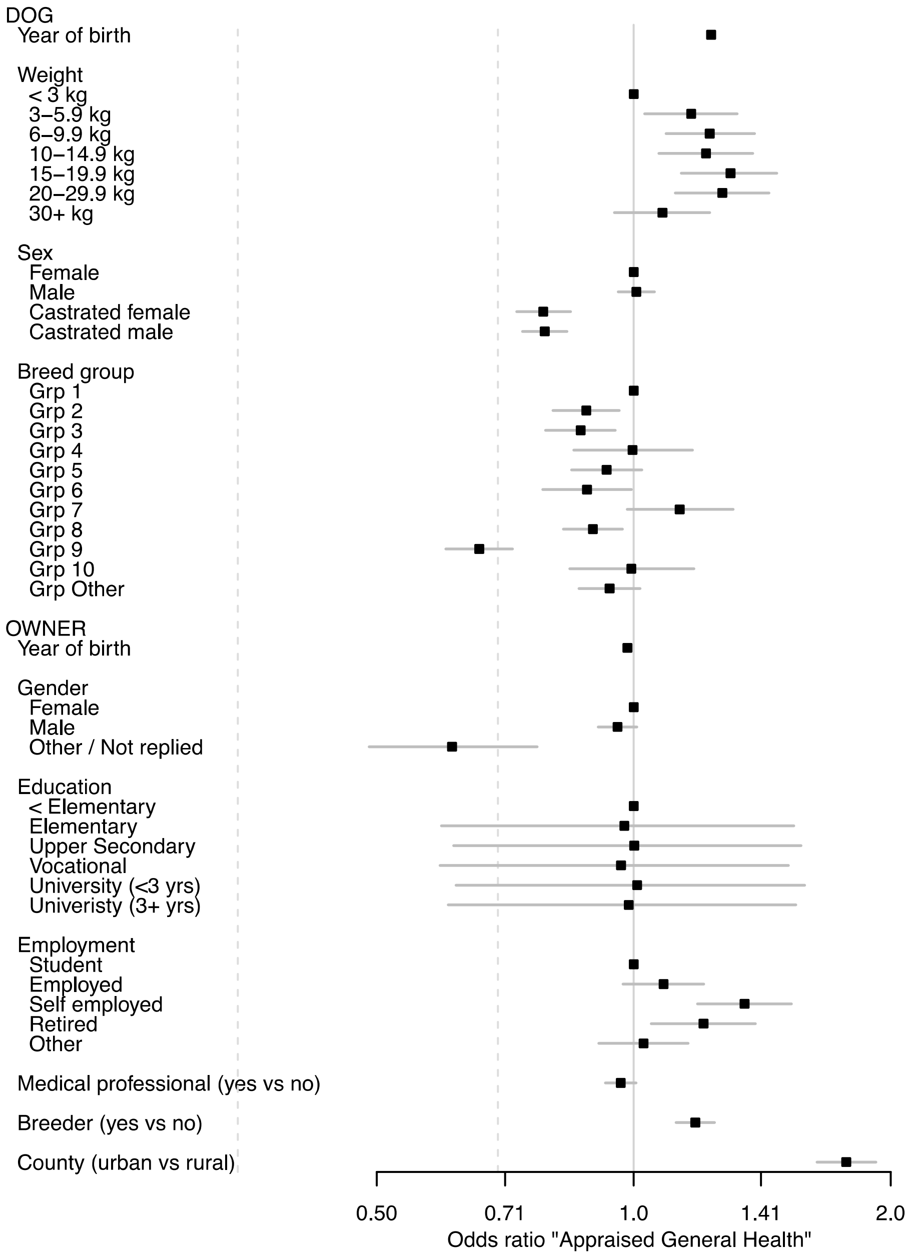

S1a. How would you appraise your dog's general health? (Q28). Odds ratio (95% CI) for dog/dog owners' background characteristics on rated general health. *Breed groups: Group 1: Sheepdogs and Cattledogs (except Swiss Cattledogs); Group 2: Pinscher and Schnauzer - Molossoid and Swiss Mountain and Cattledogs; Group 3: Terriers; Group 4: Dachshunds; Group 5: Spitz and primitive types; Group 6: Scent hounds and related breeds; Group 7: Pointing Dogs; Group 8: Retrievers - Flushing Dogs - Water Dogs; Group 9: Companion and Toy Dogs; Group 10: Sighthounds.*

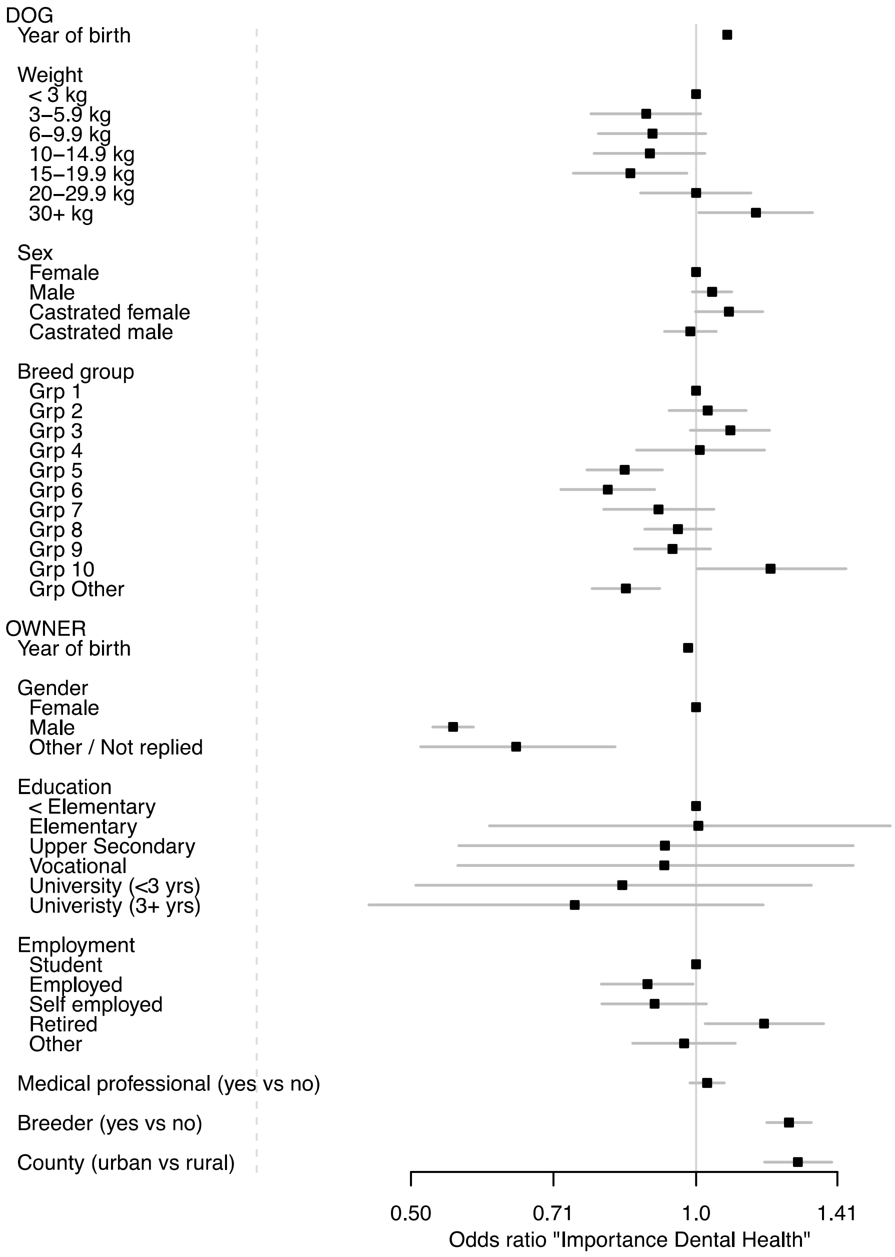

S1b. How important is it for you that your dog has good dental health? (Q6) Odds ratio (95% CI) for dog/dog owners' background characteristics on stated importance of dental health. *Breed groups: Group 1: Sheepdogs and Cattledogs (except Swiss Cattledogs); Group 2: Pinscher and Schnauzer - Molossoid and Swiss Mountain and Cattledogs; Group 3: Terriers; Group 4: Dachshunds; Group 5: Spitz and primitive types; Group 6: Scent hounds and related breeds; Group 7: Pointing Dogs; Group 8: Retrievers - Flushing Dogs - Water Dogs; Group 9: Companion and Toy Dogs; Group 10: Sighthounds.*

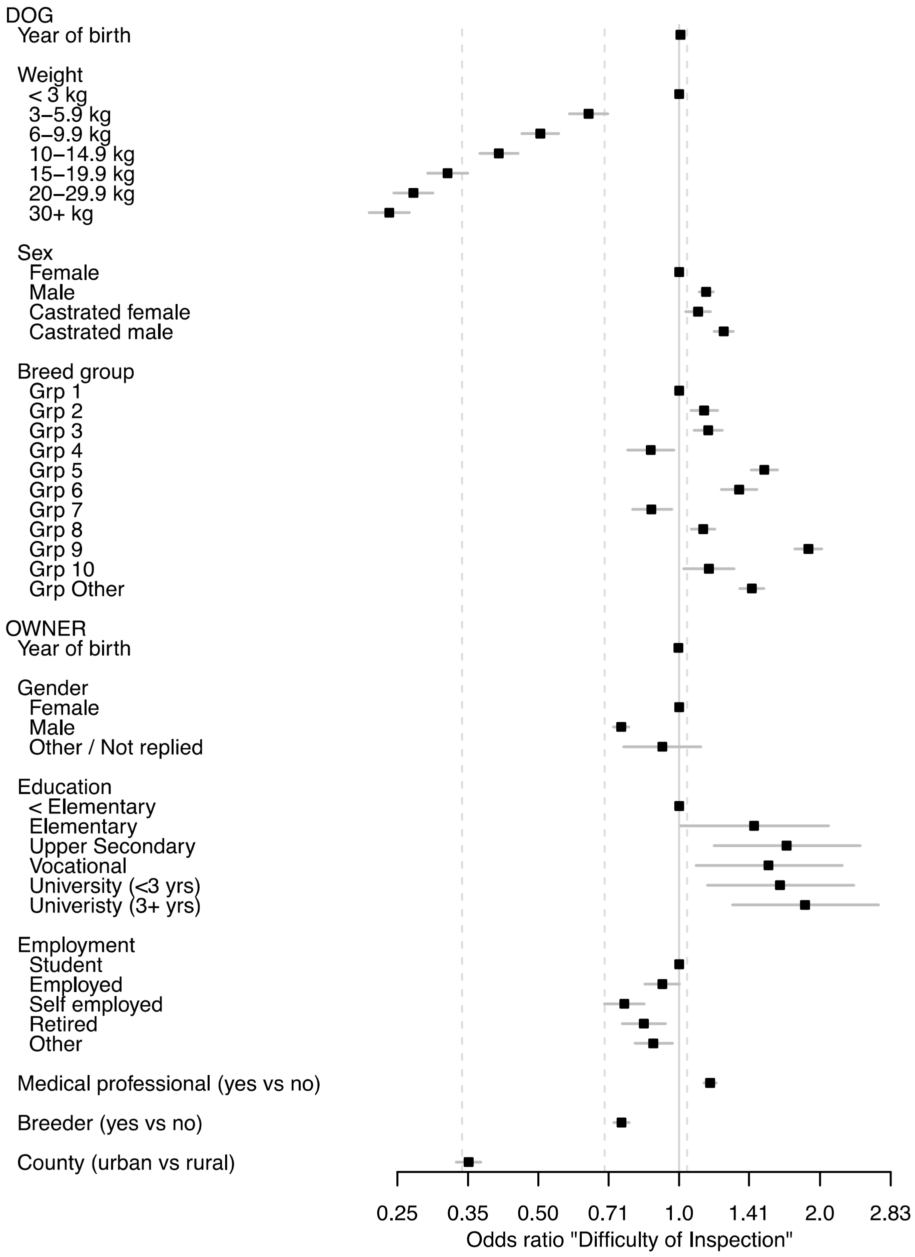

S1c. How easy or difficult is it for you to inspect (look at) all of your dog's teeth? (Q19) Odds ratio (95% CI) for dog/dog owners' background characteristics on dog owners experienced difficulties in inspecting their dog's teeth. *Breed groups: Group 1: Sheepdogs and Cattledogs (except Swiss Cattledogs); Group 2: Pinscher and Schnauzer - Molossoid and Swiss Mountain and Cattledogs; Group 3: Terriers; Group 4: Dachshunds; Group 5: Spitz and primitive types; Group 6: Scent hounds and related breeds; Group 7: Pointing Dogs; Group 8: Retrievers - Flushing Dogs - Water Dogs; Group 9: Companion and Toy Dogs; Group 10: Sighthounds.*

S1d. Concurrent diseases reported by dog owners.

**Does your dog have one or more of the following diseases: (Q29)** Several options can be specified	Diabetes (Diabetes mellitus)	35 (0.1%)
	Cushing's disease	54 (0.1%)
	Addison's disease	48 (0.1%)
	Thyroid gland disease (Hypothyroidism)	356 (0.6%)
	Heart disease	511 (0.9%)
	Kidney disease	143 (0.3%)
	Liver disease	176 (0.3%)
	Skin disease, e.g. allergy	2369 (3.9%)
	Joint disease, e.g. arthrosis	2232 (3.7%)
	None of the above mentioned diseases / Don't know	52746 (87.9%)
	Other chronic disease	2253 (3.8%)

S1e. The 10 most frequently reported breeds in the target and sample populations (relative frequency within parenthesis).

**Target**[1]	**Frequency**	**Sample**[2]	**Frequency**
Mixed Breed	236716 (27.7%)	Mixed Breed	9178 (15.3 %)
German Shepherd	29504 (3.4 %)	Labrador Retriever	2783 (4.6%)
Labrador Retriever	27416 (3.2 %)	Golden Retriever	1997 (3.3%)
Golden Retriever	23813 (2.8%)	German Shepherd	1645 (2.7%)
Chihuahua	23026 (2.7 %)	Miniature Schnauzer	1379 (2.3%)
Jack Russell Terrier	22474 (2.6 %)	Dachshund	1329 (2.2 %)
Dachshund	19554 (2.3 %)	Chihuahua	1303 (2.2%)
[No breed specified]	16590 (1.9 %)	Jack Russell Terrier	1231 (2.1%)
Swedish Elkhound	16254 (1.9 %)	Flat Coated Retriever	981 (1.6%)
Rottweiler	15529 (1.8 %)	English Cocker Spaniel	944 (1.6%)

[1]Total population of dog breeds from Swedish Board of Agricultures registry (2016). [2]Breeds as reported by dog owner respondents.

S1f. Associations of dogs' and dog owners' year of birth with owners' assessed symptoms of their dog's dental health. Higher construct score represents a relatively better perceived dental health. Scores should only be compared within figure. Note that negative scores do not automatically reflect a negative assessment of dental health.

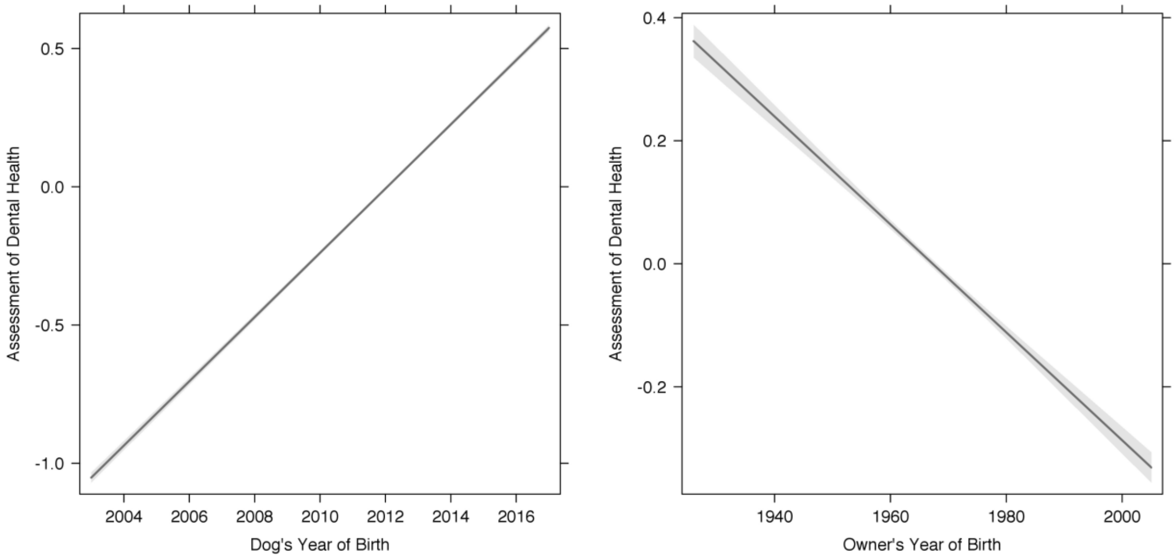

Supplement: Supplementary file 1 [file Data_Sheet_1.PDF]
